# Parental intimate partner homicide and its consequences for children: protocol for a population-based study

**DOI:** 10.1186/s12888-015-0565-z

**Published:** 2015-07-29

**Authors:** Eva Alisic, Arend Groot, Hanneke Snetselaar, Tielke Stroeken, Elise van de Putte

**Affiliations:** Monash Injury Research Institute, Monash University, 21 Alliance Lane, Clayton Campus, Melbourne, VIC 3800 Australia; Psychotrauma Centre Wilhelmina Children’s Hospital, University Medical Centre Utrecht, Lundlaan 6, Utrecht, 3584 EA The Netherlands; Wilhelmina Children’s Hospital, University Medical Centre Utrecht, Lundlaan 6, Utrecht, 3584 EA The Netherlands

**Keywords:** Bereavement, Child, Domestic violence, Femicide, Grief, Homicide, Intimate partner violence, Mental health, PTSD, Quality of life, Traumatic stress, Uxoricide, Wellbeing

## Abstract

**Background:**

The loss of a parent due to intimate partner homicide has a major impact on children. Professionals involved have to make far-reaching decisions regarding placement, guardianship, mental health care and contact with the perpetrating parent, without an evidence base to guide these decisions. We introduce a study protocol to a) systematically describe the demographics, circumstances, mental health and wellbeing of children bereaved by intimate partner homicide and b) build a predictive model of factors associated with children’s mental health and wellbeing after intimate partner homicide.

**Methods/Design:**

This study focuses on children bereaved by parental intimate partner homicide in the Netherlands over a period of 20 years (1993 – 2012). It involves an incidence study to identify all Dutch intimate partner homicide cases between 1993 and 2012 by which children have been bereaved; systematic case reviews to describe the demographics, circumstances and care trajectories of these children; and a mixed-methods study to assess mental health, wellbeing, and experiences regarding decisions made and care provided.

**Discussion:**

Clinical experience and initial research suggest that the children involved often need long-term intensive mental health and case management. The costs of these services are extensive and the stakes are high. This study lays the foundation for an international dataset and evidence-informed decision making.

## Background

At least one in seven homicides is perpetrated by an intimate partner [1]. In many cases the couple have one or more children. These children often lose both parents at once: the victim-parent is deceased and the offender-parent is detained, on the run or has committed suicide [2]. Parental intimate partner homicide often creates an absence of guardianship and involves conflict between relatives concerning children’s living arrangements. Relatives’ own grief and traumatic stress may affect their caregiving capacity, and at times, the family of the offending parent may condone the violence [3]. Changes in living arrangements can also mean that children have to change schools and lose contact with their direct social environment [4].

Concerns have been raised regarding the bereaved children’s mental health and wellbeing. In particular, Posttraumatic Stress Disorder (PTSD), strong grief reactions, and developmental difficulties have been reported [3–6]. Clinical experience suggests that many children become ‘high-end’ users of mental health and social services over multiple years, even decades. However, there are no comprehensive, systematically collected data available regarding children’s wellbeing after losing a parent due to intimate partner homicide. Most articles on the topic are clinical case studies [7, 8], case series [6, 9] or opinion pieces [10]. The long-term effects of parental intimate partner homicide, both in terms of children’s circumstances and their wellbeing, as well as the associated need for care/services are unknown.

Meanwhile, professionals – who may only be exposed to this type of case once or twice in their career – make far reaching decisions about children’s futures. These decisions regard communication about the homicide (e.g., what to tell very young children?), mental health care plans, guardianship, placement (e.g., with an unrelated family vs. with relatives of the victim or perpetrator) and contact with the perpetrating parent in prison. An evidence base to assist with these complex decisions is urgently needed.

### Research aims

To contribute to the improvement of children’s mental health and wellbeing after parental intimate partner homicide, we aim to:

1. systematically describe the demographics, circumstances, mental health and wellbeing of children bereaved by intimate partner homicide;
2. build a predictive model of factors associated with children’s mental health and wellbeing after intimate partner homicide.

The current protocol serves two purposes: a) to record the study procedures of an ongoing study in the Netherlands, and b) to serve as a template for future studies in other countries.

## Methods/Design

### Study design

We systematically study all children bereaved by parental intimate partner homicide in the Netherlands over a period of 20 years (1993 – 2012). The study has been approved by the University Medical Center Utrecht Ethics Committee (13/609, 24-12-2013). It consists of three elements:

1. *an incidence study* to identify all Dutch intimate partner homicide cases between 1993 and 2012 by which children have been bereaved;
2. *systematic case reviews* to describe the demographics, circumstances and care trajectories of these children; and
3. *a mixed-methods study* to assess mental health, wellbeing, and experiences regarding decisions made and care provided.

### Part 1: Incidence study

To identify cases of parental intimate partner homicide, we examine eight sources of data. None of these sources is fully accurate or complete; combining and cross-checking information will allow for a robust estimate of number of parental intimate partner homicides per year. The sources include: a) databases of the Child Care and Protection Board, b) databases of the national Youth Care Agency, c) databases of the Department of Justice, d) two Dutch books that describe homicide cases in the Netherlands [11, 12], and their accompanying data; e) the ‘Elsevier Murderlists’; brief descriptions of homicides published in the national magazine Elsevier; f) the client database of the Psychotrauma Centre of the Wilhelmina Children’s Hospital; g) the public database of legal verdicts (www.rechtspraak.nl); and h) the database Lexis Nexis that includes articles of all national and regional newspapers in the Netherlands. Some of the databases (e.g., the Elsevier Murderlists and the database of legal verdicts) are open to the public. For all non-public databases, we have obtained access via the relevant authorities or owners, with permission from the ethics committee. In order to guarantee reliability of the data, we do not include any cases that are mentioned in the newspapers only; cases identified in the newspapers have to be confirmed by at least one other source.

For each year (1993–2012) we make a list of intimate partner homicides in which one or more children up to 18 years of age lost a biological parent. We exclude homicides in other countries as well as cases in which the victim and/or perpetrator were not Dutch residents. We include cases based on their legal outcomes – a verdict of murder or manslaughter – with one exception: cases described as intimate partner homicide in which the perpetrator committed suicide before the verdict (often directly after the homicide) are also included. We do not include cases in which the verdict was ‘not guilty’ or in which the verdict was ‘involuntary manslaughter’. Due to the length of procedures, the children involved in some of the excluded cases will have been in very similar situations to children in included cases.

### Part 2: systematic case reviews

For each identified case we conduct a systematic review of the demographics and circumstances of the children involved. To obtain as much information as possible, we undertake three steps. First, we request available documentation from our collaborating organizations mentioned above. Second, we examine publicly available (legal) reports. Third, when children and/or caregivers participate in the mixed methods study, we ask whether they would be happy to make their case files available and/or complete information in the interviews.

For each available case we record:

- demographic characteristics of the perpetrator, victim and child(ren) (age, gender, ethnicity);
- characteristics of the family (previous domestic violence, relationship status of victim and perpetrator);
- characteristics of the homicide (weapon, witness/victim status of the children, location);
- characteristics of the legal process of the perpetrator (type of verdict, time lag between the homicide and the verdict, use of appeal processes);
- characteristics of guardian/custodianship of the child (whether the guardian is a family member, changes in guardianship over time);
- characteristics of the child’s living arrangements (with whom, for how long, whether the child had to change schools);
- characteristics of the subsequent professional social services/mental health care involved (which organizations, which services/treatment, for how long);
- characteristics of contact arrangements with the perpetrating parent (whether, when & how long there was contact);
- characteristics of contact between the family of the victim and the family of the perpetrator (the frequency of contact and whether there were conflicts); and
- children’s and families’ evaluation of the professional care/involvement.

### Part 3: mixed methods study

We collect both in-depth quantitative and qualitative information to understand current mental health and wellbeing of the children and their caregivers as well as their experiences with the decisions that have been made and the care/services that have been provided. Eligible for this part of the study are:

- children, adolescents and young adults aged 8 to 25 years who lost a parent due to intimate partner homicide when they were younger than 18 years old and
- caregivers/guardians of children, adolescents and young adults up to 25 years old who lost a parent due to intimate partner homicide.

#### Recruitment

Potential participants are recruited via three different strategies. First, based on our database from the incidence study and case reviews, participants are invited directly if they are or were clients of the Psychotrauma Centre of the Wilhelmina Children’s Hospital, a nation-wide tertiary mental health care provider. Second, if they have never been clients of the Psychotrauma Centre, we invite them via our collaborators (e.g., the Child Care and Protection Board officers) who have direct contact with them. Third, we are in contact with victim support associations and professional service associations, who notify their members and contacts of the study through newsletters and personal messages. In particular, Victim Support the Netherlands actively engages with their staff to refer potential participants. People interested in the study can approach us for further information about the study and potential participation. For all participants up to 18 years of age, we obtain written consent from the legal guardians (who may be professional guardians) before approaching the children and/or non-guardian caregivers, in collaboration with the organizations involved. Young adults can provide written consent to participate autonomously.

#### Procedures

Each young person and their caregiver/guardian are asked to:

1. fill out questionnaires on the young person’s mental health and wellbeing;
2. participate in a structured clinical interview;
3. participate in a semi-structured qualitative interview; and
4. provide information regarding any outstanding questions in the systematic case review.

We ask both the children and the caregivers/guardians to provide information because previous mental health research has shown that their reports often differ [13]. We also ask teachers of the children and adolescents to complete a questionnaire. Except for the teachers, data collection takes approximately 2.5 h per respondent. Participants have the opportunity to complete the questionnaires at their own pace at home ahead of the interview. Experienced, qualified mental health professionals (minimum Bachelor’s degree level) conduct the interviews in a quiet, confidential office space or at participants’ homes. Our staff monitor participants’ distress levels and provide support and referral if needed. Participation in the study is entirely voluntary and can be ceased at any time.

#### Materials

Our quantitative measures are shown in Table 1 and briefly described below.

**Table 1:** Structured clinical interviews and questionnaires used in the mixed methods study

Participants	Parameters	Instrument
Children (8 to 12 yrs)	PTSD and comorbid psychopathology	Anxiety Disorders Interview Schedule Child Version (ADIS-C) [14]
	Traumatic grief	Inventory of Prolonged Grief for Children (IPG-C) [27]
	Quality of life	Pediatric Quality of Life Inventory (PedsQL) [28]
Adolescents (12 to 18 yrs)	PTSD and comorbid psychopathology	Anxiety Disorders Interview Schedule Child Version (ADIS-C) [14]
	Traumatic grief	Inventory of Prolonged Grief for Adolescents (IPG-A) [27]
	Quality of life	Pediatric Quality of Life Inventory (PedsQL) [28]
	Daily functioning and behavior problems	Child Behavior Checklist – Youth Self Report (YSR) [31, 32]
Young adults (18 to 25 yrs)	PTSD and comorbid psychopathology	Structured Clinical Interview for DSM-IV-TR Axis I Disorders (SCID) [20]
	Traumatic grief	Inventory of Traumatic Grief (ITG) [24]
	Quality of life and daily functioning	RAND-36 [34, 36]
Caregivers (reporting on child/adolescent wellbeing)	PTSD and comorbid psychopathology	Anxiety Disorders Interview Schedule Parent Version (ADIS-P) [14]
	Traumatic grief	Inventory of Prolonged Grief for Children (IPG-C) Parent form [27]
	Quality of life	Pediatric Quality of Life Inventory (PedsQL) – Parents [28]
	Daily functioning and behavior problems	Child Behavior Checklist – Parent Report Form (PRF) [32, 33]
Caregivers (reporting on own & family wellbeing)	Quality of life	RAND-36 [34, 36]
	Traumatic grief	Inventory of Traumatic Grief (ITG) [24]
	Family functioning	Family Functioning questionnaire (FQ) [38]
Teachers (reporting on child/adolescent wellbeing)	Daily functioning and behavior problems	Child Behavior Checklist – Teacher Report Form (TRF) [32, 33]

#### Anxiety disorders interview schedule for children (ADIS)

The ADIS [14, 15] is a semi-structured interview focusing on anxiety disorders including PTSD, according to the DSM-IV-TR criteria. It also allows for screening of affective disorders, externalizing behavior, use of substances, and eating disorders. The interview has both a child version (ADIS-C; for children aged 7 and older) and a caregiver version (ADIS-P). The reliability and validity of the ADIS-C and ADIS-P have been tested extensively, with positive results [14–19].

#### Structured clinical interview for DSM disorders (SCID)

The SCID [20, 21] is a semi-structured interview for the assessment of the major DSM-IV Axis I diagnoses among adults. Besides PTSD, it examines symptoms of other anxiety disorders, mood disorders, psychotic disorders, substance use disorders, somatoform disorders and eating disorders. We use this interview for the assessment of the young adults who have been bereaved as children. The SCID has been shown to be valid and reliable [21–23] and is one of the most widely used structured clinical interviews internationally.

#### Inventory of traumatic grief (ITG)

The ITG [24, 25] is a 30-item questionnaire to measure maladaptive symptoms of grief. The measure is an expanded version of the Inventory of Complicate Grief [26]. The Dutch version of the ITG has been shown to be highly reliable (Cronbach’s α 0.96) and valid [25].

#### Inventory of prolonged grief – Children/Adolescents (IPG)

The IPG-C and IPG-A are Dutch questionnaires to assess symptoms of Prolonged Grief Disorder in children aged 8 to 12 years and adolescents aged 13 to 18 years [27]. The questionnaire consists of 30 items referring to the experience of psychological, psychosomatic and behavioral symptoms related to the death of a loved one. Internal consistency for both versions is good (Cronbach’s α of 0.91 and 0.94), there is support for a one-factor structure of the measures and their convergent validity have been established [27]. Since the original measures were for the young participants only, we have developed a caregivers’ version in collaboration with the authors, maintaining the exact content of the 30 items while rephrasing items to “my child…” instead of “I…”.

#### Pediatric quality of life inventory (PedsQL)

The PedsQL [28, 29] uses a modular approach to measuring health-related quality of life and includes 22 questions, divided into four scales: physical functioning, emotional functioning, social functioning, and school functioning. The questionnaire has a self-report version for children and adolescents as well as a caregiver report version. The reliability for both child and parent report have shown to be excellent in international research (Cronbach’s α of 0.89 and 0.92 respectively [28]) and has been confirmed as good (0.82 to 0.85) in the Dutch context [30].

#### Child behavior checklist (CBCL)

The CBCL [31, 32] is a child behavior questionnaire completed by parents (PRF), teachers (TRF) and/or the child (YSR). All versions are used in this study. The questionnaire includes 20 competence items and 100 to 118 specific problems items on a three-point-scale. The CBCL measures behavioral problems in eight subscales such as ‘Social Problems’ and ‘Attention Problems’, within two combined scales of internalizing and externalizing behavior. The measure has shown adequate reliability and validity [33] and is used widely. The internal consistence of the measure is good (Cronbach’s α of the total scale is 0.97 for the PRF, 0.95 for the YSR and 0.97 for the TRF) as is its test-retest reliability (ICCs varying from 0.87 to 0.95) [33].

#### RAND-36

The RAND-36 [34, 35] is a short form questionnaire based on the RAND Health Insurance Study Questionnaire [36]. It measures quality of life in adults through nine subscales, such as physical functioning, social functioning, mental health, and general health perception. The measure consists of 11 questions with multiple sub questions, on two-point to six-point scales. In Dutch studies, reliability and validity of the scale have shown to be adequate. Internal consistency is moderate to good (Cronbach’s α varying from 0.71 to 0.92) and the test-retest correlations are sufficient (ICC varying from 0.58 to 0.82 after two months) [35, 37].

#### Family questionnaire (FQ)

The FQ [38] is a Dutch questionnaire which assesses family functioning in families with children aged 4 to 18 years old, through caregiver report. It includes 45 items on five scales: responsiveness, communication, organization, partner relationship and social network. The reliability of the measure is moderate to excellent (Cronbach’s alpha varying from .79 to .95 for subscales and .97 for the total scale) and test-retest correlations are good (ICC for the total score is .91 and the ICC for subscales is varying from 0.78 to 0.89). The validity, including criterion, convergent and discriminant validity (against the Family Environment Scales; [38]), has also been confirmed [39].

#### Qualitative interviews

For the semi-structured qualitative interviews for both children and caregivers, we use a ‘topic list’ [40]. The questions are adapted to the young person’s developmental level and cover experiences related to psychosocial development, placement, contact with the perpetrating parent, custody/guardianship, the role of relatives, the role of professional organizations, helping factors, and contact with people who have also lost family due to homicide, as well as expectations of the future. When necessary, follow-up questions are posed. In addition, there is an opportunity for the participants to raise issues themselves. All interviews are audiotaped.

### Analyses

Our main analyses will be quantitative and will describe the circumstances of the children who have been bereaved by intimate partner homicide. This will predominantly involve frequencies, means, and proportions based on the incidence study and the systematic case reviews. For psychological variables derived from the mixed-methods study we will indicate whether total scores are significantly higher, lower, or equivalent to the available norms for healthy and/or clinical populations. When relevant (e.g., when describing characteristics of the perpetrator and victim), statistics will be reported at family level, to avoid interdepence of information on children within families. Regression analyses and analyses of variance to examine the associations between potential predictors (e.g., prior domestic violence, witnessing the homicide, contact with the perpetrator, placement with an unrelated family vs. with relatives of the victim or perpetrator) and outcomes (e.g., PTSD status, quality of life) will be exploratory (see also below regarding sample sizes). For our qualitative analysis, all interviews will be transcribed verbatim, with names substituted with functional codes. We will use a framework approach involving the phases of familiarization, identification of a thematic framework, indexing, charting, and mapping and interpretation [41].

#### Sample size

Sample sizes for this study are limited by the relatively low incidence of intimate partner homicides. We aim to involve all cases from 1993 to 2012, estimated at 260 families and 520 children. Our goal is to obtain 60 % of the cases in the case file reviews and 15 % in the mixed methods study. For any regression analyses and analyses of variance, we will aim for a maximum of 1 variable per 10 cases in the analysis under consideration. Since previous research on children bereaved by intimate partner homicide is lacking, effect sizes are unknown. However, describing the population with frequencies and means will be possible, and our design allows for an estimate of participation bias in the mixed methods study. To allow more robust analyses in the future, we ask participants permission to combine their de-identified data with data from replications in other countries in the future. Regarding the qualitative analyses, the sample sizes should be sufficient to reach saturation of themes [40].

## Discussion

There is an urgent need for an evidence base regarding children’s mental health and psychosocial wellbeing after parental intimate partner homicide. Clinical experience and initial research suggest that the children involved often need long-term intensive mental health and social services/case management. The costs of these services are extensive [42] and the stakes are high, in part also due to the cases’ high media profile. Within a short timeframe, professionals have to make far-reaching decisions regarding communication about the homicide (e.g., what to tell very young children?), custody arrangements, living arrangements (e.g., placement with the family of the victim, the perpetrator, or a foster family), mental health treatment, and contact arrangements with the perpetrating parent. More knowledge, leading to guiding principles to facilitate these decisions, is therefore required. While this project focuses on the unique situation of children bereaved by intimate partner homicide, it will also inform other areas of extreme child trauma, such as witnessing the homicide of a family member in other circumstances. Once the database is established, it will provide the foundation for longitudinal monitoring of children to better understand which factors shape their outcomes. Moreover, in our view, it will be necessary to go beyond a national perspective. The current protocol can be applied to other countries in order to create an international dataset. This will allow more specific and robust analyses, as well as facilitate an understanding of the role of legal and social differences across countries. Over time, the creation of an international dataset will spur the development of well-grounded practice guidelines.

Dissemination of the study’s findings will include articles in peer-reviewed journals as well as workshops with practitioners and policy makers to discuss interpretation of the results and implications for policy and practice. With the aim of improving the circumstances and outcomes of the children involved, it will be crucial to take into account both quantitative and qualitative accounts on what helps them navigate the accumulation of challenges they are often confronted with.

## Abbreviations

ADIS: Anxiety disorders interview schedule for children
ADIS-C: Anxiety disorders interview schedule child version
ADIS-P: Anxiety disorders interview schedule parent version
CBCL: Child behavior checklist
PRF: Child behavior checklist – parent report form
TRF: Child behavior checklist – teacher report form
YSR: Child behavior checklist – youth self report
FQ: Family functioning questionnaire
IPG: Inventory of prolonged grief
IPG-A: Inventory of prolonged grief for adolescents
IPG-C: Inventory of prolonged grief for children
ITG: Inventory of traumatic grief
PedsQL: Pediatric quality of life inventory
PTSD: Posttraumatic stress disorder
SCID: Structured clinical interview for DSM-IV-TR Axis I Disorders
